# 891 Skin Is In: A National Analysis of Skin Cell Suspension Autograft in Burn Centers

**DOI:** 10.1093/jbcr/iraf019.422

**Published:** 2025-04-01

**Authors:** Athena Hoppe, Jeffrey Carter, Bart Phillips, Herbert Phelan, Jonathan Schoen, Anastasiya Ivanko, Victoria Miles

**Affiliations:** Louisiana State University; University Medical Center New Orleans; BData, Inc; University Medical Center New Orleans; Louisiana State University Health Sciences Center, New Orleans; University Medical Center New Orleans; University Medical Center New Orleans

## Abstract

**Introduction:**

In an effort to improve patient care and burn/blast disaster preparedness, the Administration for Strategic Preparedness and Response’s Biomedical Advanced Research Development Authority has invested over $1 billion in burn and blast research over the last 10 years advancing many treatment paradigms. These innovations require effective and equitable implementation into clinical practice to meet the intended purpose. Our goal was to examine the use of skin cell suspension autograft in burn care over the last 5 years.

**Methods:**

Following IRB approval and data use agreements, the American Burn Association’s (ABA) Burn Care Quality Platform (BCQP) was queried for autologous skin cell suspension (ASCS) autograft cases among admissions by reporting burn centers from 01/2019-06/2024. Aggregate data comprising of injury volume, etiology, location, severity, demographics, disposition, modified Baux score, length of stay, wound closure rates, and admission diagnosis related groups (DRGs) were collected. Analysis was performed in partnership with BData Inc. using proprietary software.

**Results:**

6,310 burn and/or trauma admissions were identified in BCQP as utilizing ASCS with a 3.3% mortality rate. Patients < 16 years of age represented 11.1%. The most common burn etiologies were flame (51.9%), scald (22.9%), and flash (11.4%). As for burn injury severity, 30.6% had a < 10% TBSA and 28.0% with a 10-19% TBSA. The median modified Baux score 62.5 (SD 28.8). Arms (80.2%), trunk (66.3%), legs (65.8%), and head/face (47.4%) were the most common sites of injury. The 3 largest payor classes were Medicaid (33.3%), Private/Commercial (27.1%), and Medicare (16.6%). Disposition was home in 66.0% of cases. The top 3 DRGs were 928 (1,933 admissions), 927 (1,125 admissions), and 929 (939 admissions). Median LOS was 11.0 days for 0-10% TBSA (n=1,932, SD 24.6), 17.0 days for 10-19% TBSA (n=1,768, SD 21.0), and 27.0 for 20-29% TBSA (n=942, SD 23.5). Median wound closure prior to discharge was >90% (n=2,755, SD 37.5).

**Conclusions:**

This study is the largest to date to examine ASCS use. ASCS was utilized in burn care across varying demographics, payors, and burn severities with a noted annual increases in case volume. At over 6,000 cases in 5 years, BCQP offered unprecedented detail of surgical technique and outcomes to support new insights. Additional studies should compare outcomes with concurrent controls to guide further innovation, reimbursement, and burn clinical care.

**Applicability of Research to Practice:**

ASCS is a new technology that is not utilized in all burn centers. This study is the largest analysis of real-world data of ASCS used in burn care and will aid in guiding future research efforts and clinical practices.

**Funding for the Study:**

N/A

